# Pioneering *BRCA1/2* Point-Of-Care Testing for Integration of Germline and Tumor Genetics in Breast Cancer Risk Management: A Vision for the Future of Translational Pharmacogenomics

**DOI:** 10.3389/fonc.2021.619817

**Published:** 2021-09-29

**Authors:** Lwando Mampunye, Nerina C. van der Merwe, Kathleen A. Grant, Armand V. Peeters, Rispah Torrorey-Sawe, David J. French, Kelebogile E. Moremi, Martin Kidd, Petrus C. van Eeden, Fredrieka M. Pienaar, Maritha J. Kotze

**Affiliations:** ^1^Division of Chemical Pathology, Department of Pathology, Faculty of Medicine and Health Sciences, Stellenbosch University, Cape Town, South Africa; ^2^Department of Biomedical Sciences, Faculty of Health and Wellness, Cape Peninsula University of Technology, Cape Town, South Africa; ^3^Division of Human Genetics, National Health Laboratory Service, Universitas Hospital, Bloemfontein, South Africa; ^4^Division of Human Genetics, Faculty of Health Sciences, University of the Free State, Bloemfontein, South Africa; ^5^Immunology Department, School of Medicine, College of Health Sciences, Moi University, Eldoret, Kenya; ^6^Division of Health Science and Innovation, LGC Limited, Teddington, United Kingdom; ^7^Centre for Statistical Consultation, University of Stellenbosch, Stellenbosch, South Africa; ^8^Oncology Practice, Durbanville Mediclinic, Cape Town, South Africa; ^9^CancerCare, Panorama Mediclinic, Cape Town, South Africa; ^10^Division of Chemical Pathology, National Health Laboratory Service, Tygerberg Hospital, Cape Town, South Africa

**Keywords:** breast cancer, *BRCA1*, *BRCA2*, Africa, point-of-care, first-tier genetic testing, pathology, pharmacogenomics

## Abstract

Research performed in South African (SA) breast, ovarian and prostate cancer patients resulted in the development of a rapid BRCA point-of-care (POC) assay designed as a time- and cost-effective alternative to laboratory-based technologies currently used for first-tier germline DNA testing. In this study the performance of the new assay was evaluated for use on a portable screening device (ParaDNA), with the long-term goal to enable rollout at POC as an inventive step to meet the World Health Organization’s sustainable development goals for Africa. DNA samples for germline testing were obtained retrospectively from 50 patients with early-stage hormone receptor-positive breast cancer referred for genomic tumor profiling (MammaPrint). Currently, SA patients with the luminal-type breast cancer are not routinely selected for *BRCA1/2* testing as is the case for triple-negative disease. An initial evaluation involved the use of multiple control samples representing each of the pathogenic founder/recurrent variants included in the BRCA 1.0 POC Research Assay. Comparison with a validated laboratory-based first-tier real-time polymerase chain reaction (PCR) assay demonstrated 100% concordance. Clinical utility was evident in five patients with the founder *BRCA2* c.7934delG variant, identified at the 10% (5/50) threshold considered cost-effective for *BRCA1/2* testing. *BRCA2* c.7934delG carrier status was associated with a significantly younger age (p=0.03) at diagnosis of breast cancer compared to non-carriers. In three of the *BRCA2* c.7934delG carriers a high-risk MammaPrint 70-gene profile was noted, indicating a significantly increased risk for both secondary cancers and breast cancer recurrence. Initiating germline DNA testing at the POC for clinical interpretation early in the treatment planning process, will increase access to the most common pathogenic *BRCA1/2* variants identified in SA and reduce loss to follow-up for timely gene-targeted risk reduction intervention. The ease of using cheek swabs/saliva in future for result generation within approximately one hour assay time, coupled with low cost and a high *BRCA1/2* founder variant detection rate, will improve access to genomic medicine in Africa. Application of translational pharmacogenomics across ethnic groups, irrespective of age, family history, tumor subtype or recurrence risk profile, is imperative to sustainably implement preventative healthcare and improve clinical outcome in resource-constrained clinical settings.

## Introduction

Breast cancer (BC) is a leading cause of cancer among women globally, with poor survival and higher mortality rates reported in Africa. These are generally ascribed to late-stage presentation and a delay in diagnosis, partly due to sub-optimal healthcare systems (1–3). From studies conducted in Sub-Saharan Africa, more advanced breast disease is seen in patients living in rural areas than those in urban centers (2, 4). This is also the case for South Africa (SA), where the stage of cancer and age at diagnosis differs according to geographic location as well as psychosocial and personal financial status (5, 6). Fear of dying from cancer or refusal of recommended medical treatment methods due to cultural beliefs are all factors affecting overall survival (7). Conversely, should patients agree to undergo therapy, the costs related to follow-up visits may be unsustainable. Lack of community awareness relating to genetic testing and the benefits of presymptomatic diagnosis of BC contribute to the increased mortality (3).

Epidemiological studies have indicated multiple risk factors associated with the development of BC, both modifiable and non-modifiable. The influence of modifiable factors on BC risk can be controlled and is associated with lifestyle and the environment, for example, obesity and alcohol consumption (8, 9). Non-modifiable risk factors include sex, age, and age at menarche (10, 11). Menarche before the age of 12 and menopause after age 55 prolong the time that breast tissue is exposed to hormonal influence and increase the risk of BC. Genetic risk factors for cancer development or recurrence play a prominent role, especially in the presence of a family history of the disease in first-degree or multiple relatives, and a personal history of atypical hyperplasia or carcinoma *in situ* of the breast. Radiation therapy to the chest area for other malignancies before the age of 30, especially if the patient is left with intact ovarian function for ≥20 years post-treatment, may also increase the risk of BC (12).

Translational research performed in SA involving the highly penetrant *BRCA1* and *BRCA2* cancer susceptibility genes has identified various recurrent and founder variants as targets for both pharmacogenetic and cascade testing across population groups (13–17). The present article is the second in a series initiated by Oosthuizen et al. (18), aimed at the development of practical solutions for the challenges currently experienced with implementation of genomic medicine in Africa. The authors provided an historical view on *BRCA1/2* testing performed in nearly 2000 breast/ovarian cancer patients extending from a first-tier *BRCA1/2* population-based assay to next-generation sequencing (NGS) in a subset of patients. Detection of founder/recurrent variants in the majority (74%) of SA patients justified the use of a first-tier assay to select patients eligible for NGS of the *BRCA1/2* or other cancer susceptibility genes. However, uptake of laboratory-based *BRCA1/2* testing in affected families was relatively low, despite the knowledge that gene-targeted therapy and surgical intervention could be life-saving. These findings provided a strong incentive for development of a novel point-of-care (POC) test kit (https://gtr.ukri.org/projects?ref=103993) including eight of the pathogenic founder/recurrent variants previously identified in SA (17): *BRCA1* c.68_69delAG (rs80357914), c.1374delC (rs397508862), c.2641G>T (rs397508988), c.5266dupC (rs80357906)] and *BRCA2* c.5771_5774delTTCA (rs80359535), c.5946delT (rs80359550), c.6447_6448dupTA (rs397507858), c.7934delG (rs80359688). A risk-benefit analysis showed strong support (94%) for clinical implementation of a BRCA POC assay as a rapid first-tier test combined with genetic counseling (18). Implementation of our pathology-supported genetic testing (PSGT) strategy will enable *BRCA1/2* screening in BC patients unselected by age or family history through integration of germline DNA testing with tumor gene profiling, as envisaged for future application of pharmacogenomics in Africa. The cost-saving PSGT approach was first implemented in SA to reduce chemotherapy overtreatment as informed by multi-gene expression profiling (MammaPrint) (19) and to facilitate reclassification of early-stage BC into treatment groups by combining immunohistochemistry (IHC) assessment at the protein level with molecular subtyping (20), using formalin fixed paraffin embedded (FFPE) tumor biopsies. Germline DNA testing and tumor genetics based on RNA analysis are not routinely integrated to facilitate differential diagnosis and recurrence risk assessment in the same patient.

As a targeted genetic testing approach proved valuable as a first-tier test in the age of low-cost NGS (18), this study aimed to evaluate the BRCA 1.0 POC Research Assay as a robust, cost-effective alternative to currently-used laboratory-based testing protocols in BC patients unselected by family history. To our knowledge, *BRCA1/2* POC testing is not currently available internationally either as a stand-alone test or incorporated into the PSGT framework (17–20). Once assessed in relevant clinical settings, this cost- and time-effective genetic testing approach using DNA obtained from crude saliva, mouth swabs, or blood samples in conjunction with parallel genetic counseling, may be presented as a model to the policymakers at the SA Department of Health for rollout in primary health clinics. The benefits of transferring a laboratory-based assay (requiring sample transport and batching) to a rapid assay performed at POC would be three-fold: (i) to alleviate the financial burden of genetic testing in the country by identifying the most common founder/recurrent *BRCA1/2* variant carriers early using cost-effective rapid POC technology; (ii) increase healthcare accessibility of all citizens, and (iii) contribute to community awareness and education by simultaneously explaining the value of pharmacogenomics and presymptomatic diagnosis in high-risk families.

## Materials and Methods

This study included 50 patients and ten control individuals. DNA samples for germline DNA testing were obtained with written informed consent from a subset of SA patients previously referred for transcriptional gene profiling (MammaPrint/BluePrint) using FFPE tumor biopsies (19, 20). The study cohort was selected retrospectively based on a personal history of BC and IHC assessment of estrogen receptor (ER), progesterone receptor (PR), and human epidermal growth factor receptor-2 (HER2) status incorporated into the PSGT framework. The specific selection criteria for germline *BRCA1/2* POC DNA testing were different from conventional germline *BRCA1/2* testing, as it was not based on the age at onset, the presence of a family history of cancer and/or triple-negative disease (17, 21). Ethics approval was obtained from both the Health and Wellness Sciences Research Ethics Committee of the Cape Peninsula University of Technology in Cape Town (CPUT/HW-REC 2018/H10), and the Ethics Committee of the Faculty of Health Sciences, University of the Free State (UFS-HSD2019/1835/291001). The research was also approved as a sub-study under reference number N09/06/166 by the Health and Research Ethics Review Committee of Stellenbosch University, SA.

**Figure 1:**
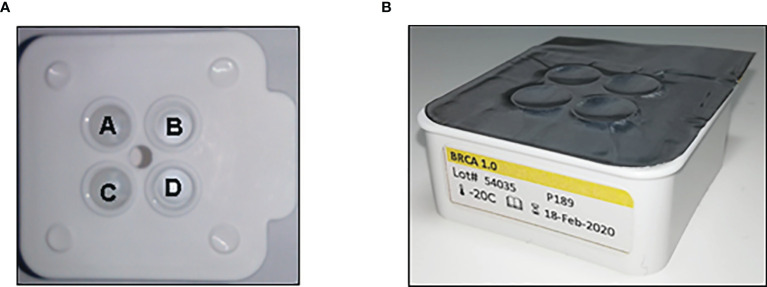
The ParaDNA reaction plate with positions of wells A–D as indicated **(A)**. The probe mixes for *BRCA1* c.1374delC (rs397508862) and *BRCA2* c.7934delG (rs80359688) are multiplexed in well A, with mixes for *BRCA1* c.2641G>T (rs39750888) and *BRCA2* c.5771_5774del (rs80359535) in well B, *BRCA1* c.5266dupC (rs80357906) and c.68_69delAG (rs80357914) in well C and *BRCA2* c.6447_6448dupTA (rs397507858) and c.5946delT (rs80359550) loaded in well D. **(B)** The ParaDNA reaction plates are provided foil sealed, ready for use.

The BRCA 1.0 POC Research Assay and instrumentation were provided by the LGC Limited (Teddington, UK), using HyBeacon probes synthesized by LGC, Biosearch Technologies (Petaluma, USA). Kit development by LGC was based on the ParaDNA polymerase chain reaction (PCR) amplification principles as previously described (22, 23). The reaction plate kits (BRCA 1.0) were stored at -20°C and thawed at room temperature for 15–20 min before use. DNA samples were diluted to a final concentration of 1 ng/ul, and 2 µl of each sample transferred into each well of the ParaDNA reaction plate (**Figure 1**). The ParaDNA assay comprised all the reagents required for multiplex melt curve analysis of eight *BRCA1/2* targets in a four-tube format (**Table 1**) using the fluorescent dyes FAM, CAL Fluor Orange 560 (CAL560), and CAL Fluor Red 610 (CAL610).

**Table 1:** Multiplex analysis of eight *BRCA1/2* founder/recurrent variants in a four-tube ParaDNA closed system format.

Well	Gene	Founder/recurrent variant^a^	Variant	Probe label
**A**	*BRCA1*	c.1374delC	rs397508862	FAM
	*BRCA2*	c.7934delG	rs80359688	CAL560
**B**	*BRCA1*	c.2641G>T	rs397508988	FAM
	*BRCA2*	c.5771_5774del TTCA	rs80359535	CAL560
**C**	*BRCA1*	c.5266dupC	rs80357906	FAM
	*BRCA1*	c.68_69delAG	rs80357914	CAL610
**D**	*BRCA2*	c.6447_6448dupTA	rs397507858	FAM
	*BRCA2*	c.5946delT	rs80359550	CAL560

a Reference sequences used for BRCA1 and BRCA2 analyses were GenBank NM_007294.4 (BRCA1) and NM_000059.3 (BRCA2).

**Figure 2:**
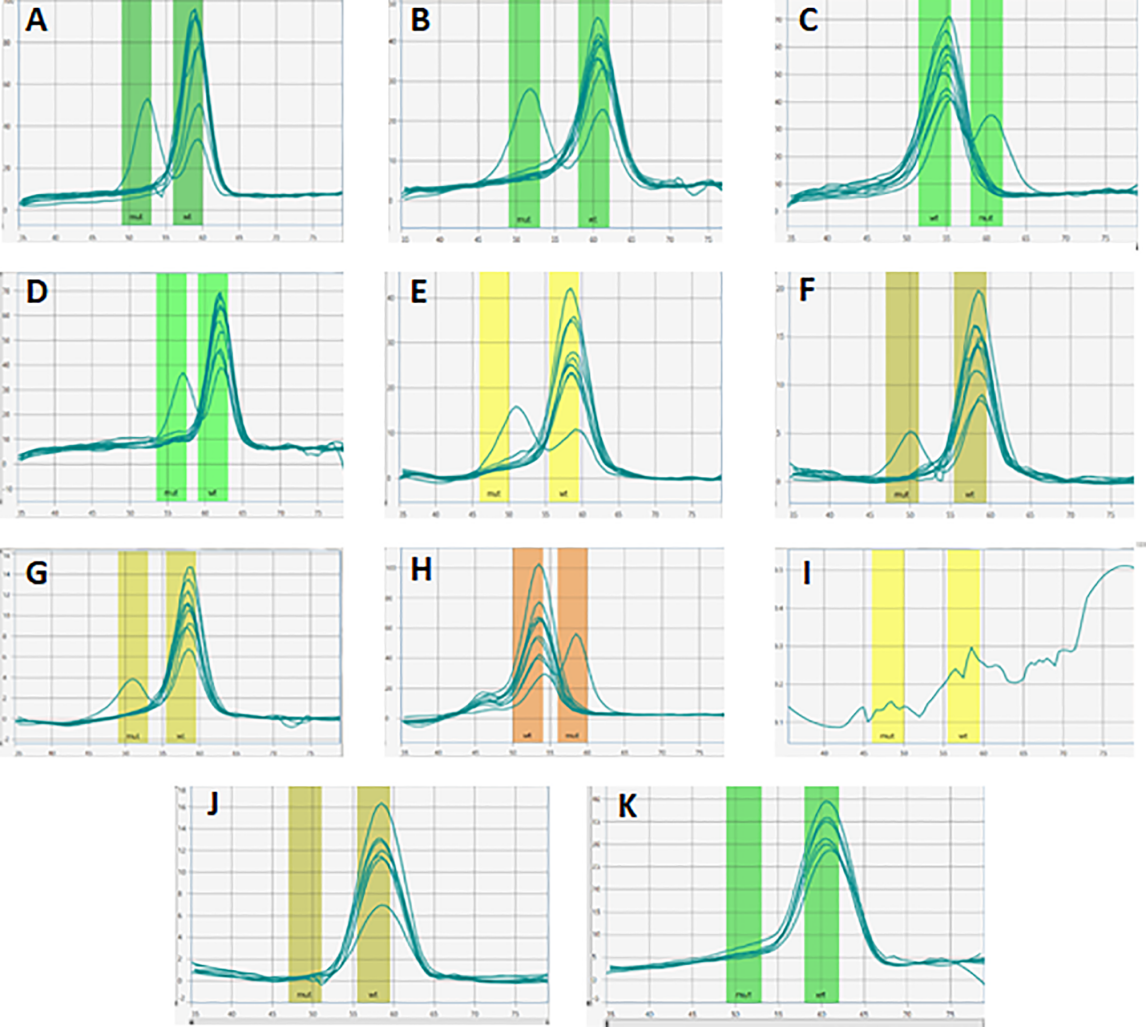
*BRCA1/2* genotyping of control samples using the BRCA 1.0 POC assay. Melting curve analysis of founder mutations *BRCA1* c.68_69delAG, c.1374delC, c.2641G>T, c.5266dupC and *BRCA2* c.5771_5774delTTCA, c.5946delT, c.6447_6448dupTA, c.7934delG **(A–H)**. By using the HyBeacon FAM probe (green), melting peaks were correctly detected for pathogenic variants *BRCA1* c.1374delC (rs397508862) **(A)**, *BRCA2* c.6447_6448dupTA (rs397507858) **(B)**, *BRCA1* c.2641G>T (rs397508088) **(C)**, *BRCA1* c.5266dupC (rs80357906) **(D)**, while the CAL 560 probe (orange) detected *BRCA2* c.5771_5774delTTCA (rs80359535) **(E)**, *BRCA2* c.5946delT (rs80359550) **(F)**, *BRCA2* c.7934delG (rs80359688) **(G)** and the CAL 610 probe identified the pathogenic variant *BRCA1* c.68_69delAG (rs80357914) **(H)** with fluor red dye on the controls with known *BRCA1/2* variants. No peaks were detected in **(I)**, confirming the absence of amplification in the blank sample containing no template DNA. The absence of a second melting curve in **(J)** and **(K)** was expected and confirmed the negative controls as samples without any specific founder/recurrent pathogenic variants.

Prior to the analysis of the 50 patient samples using DNA extracted from whole blood and/or saliva, a no template control and two *BRCA1/2* variant-negative controls, as well as eight variant-positive samples of known genotype were tested. The genotypes of the DNA samples used as positive controls were previously determined using a combination of validated hybridization and simple probe technologies (18). Negative controls were previously screened using NGS based on standard selection criteria for *BRCA*/other high-moderate penetrance cancer susceptibility genes (17). The accuracy of the genotyping calls was assessed by adding different DNA samples representing the known SA founder/recurrent variants to each well of the ParaDNA plates. The plates were inserted into the ParaDNA instrument for rapid thermal cycling. Following an initial denaturation step (98°C for 1 min), the targets were amplified using 50 PCR cycles of 99°C for 7 sec, 62°C for 12 sec and 72°C for 12 sec, followed by denaturation at 95°C for 20 sec and probe annealing at 35°C for 30 sec. Melting curve analysis was performed by heating the samples from 35°C to 80°C using a 0.1°C/sec ramp rate and fluorescence acquisition. The ParaDNA software (version 1.6.0.27) automatically analyzed the melting curves and computed the associated *BRCA1/2* genotypes (**Figures 2A–K**) within approximately one hour. Automated software calls were assessed using the ParaDNA Data Review software to examine sample melting curves. Once the BRCA 1.0 Research Assay’s performance was confirmed using the specific controls, germline DNA of 50 hormone receptor-positive BC patients previously analyzed with the 70-gene MammaPrint assay, was genotyped. Reference sequences used for *BRCA1* and *BRCA2* analyses were GenBank NM_007294.4 (*BRCA1*) and NM_000059.3 (*BRCA2*).

The genotyping calls generated by the BRCA 1.0 Research Assay were confirmed using alternative methods including real-time PCR conducted by means of the Roche LightCycler hybridization and simple assay systems (18) and/or Sanger sequencing using the primer sets listed in **Table 2** and the BigDye^®^ Terminator v3.1 Cycle Sequencing Kit (Thermo Scientific Corp., Waltham, MA) on an ABI Genetic Analyzer. The electropherograms were analyzed by visual inspection and aligned to the reference sequences.

**Table 2:** Oligonucleotide primers used for conventional polymerase chain reaction application and Sanger sequencing of *BRCA1* (NM007294.4) and *BRCA2* (NM000059.3) gene regions spanning the nucleotide positions of eight pathogenic founder/recurrent mutations previously identified in the multi-ethnic South African population.

Gene	Region	Variant	Primer^a^	Oligonucleotide primers (5’ to 3’)	Size (bp)
*BRCA1*	Exon 2	c.68_69delAG p.(Glu23ValfsX17)	F	TGTGTTAAAGTTCATTGGAACA	149
		[Jewish, European]	R	CATAGGAATCCCAAATTAATACA	
	Exon 10	c.1374delC (p.Asp458GlufsX17)	F	TCGCATGCTCAGAGAATCC	400
		[Afrikaner]	R	TGTGGCTCAGTAACAAATGCTC	
	Exon 10	c.2641G>T (p.Glu881Ter)	F	GCTCAGTATTTGCAGAATAC	253
		[Afrikaner]	R	GCTTATCTTTCTGACCAACC	
	Exon 19	c.5266dupC (p.Gln1756ProfsX74)	F	AGTCAGAGGAGATGTGGTCAATGG	236
		[Ashkenazi Jewish]	R	GTGGTTGGGATGGAAGAGTGAA	
*BRCA2*	Exon 11	c.5946delT (p.Ser1982ArgfsX22)	F	CGAGGCATTGGATGATTCAGAG	394
		[Ashkenazi Jewish]	R	GAGCTGGTCTGAATGTTCGTTAC	
		c.6447_6448dupTA (p.Lys2150IlefsX19)	F	GAGAAACCCAGAGCACTGTG	404
		[Mixed Ancestry]	R	CTAAGATAAGGGGCTCTCCTC	
		c.5771_5774del TTCA (p.Ile1924ArgfsX38)	F	CGAGGCATTGGATGATTCAGAG	394
		[Xhosa, Mixed Ancestry]	R	GAGCTGGTCTGAATGTTCGTTAC	
	Exon 17	c.7934delG (p.Arg2645AsnfsX3)	F	GTAGTTGTTGAATTCAGTATC	354
		[Afrikaner, Mixed Ancestry]	R	TGGCAACTGTCACTGACAAC	

a F, forward; R, reverse.

The data were analyzed and described using cross-tabulation and frequency tables analyzed using the STATISTICA package. One-way ANOVA was used to compare the average age between subgroups. The significance level was set at 0.05 for the determination of statistical significance.

## Results

The BRCA 1.0 POC Research Assay was first standardized in the laboratory by using ten control samples before commencing testing of the study cohort. These samples represented each of the eight selected *BRCA1/2* founder/recurrent SA pathogenic variants, evaluated together with a no template and two variant-negative controls. All samples were genotyped using 3-color, 4-tube multiplex assays after adding the extracted DNA to each of the four plate wells. The test duration from sample-to-result was approximately one hour, excluding previously performed DNA extraction and quantification. The ParaDNA software automatically analyzed the multiplex melt curve data and generated genotype calls (**Figures 2A–K**). There was a 100% concordance between the genotyping calls obtained by the ParaDNA instrument and software and those identified using alternative methods. All the samples and negative controls were correctly assigned using 2 ng of input DNA only.

**Figure 3:**
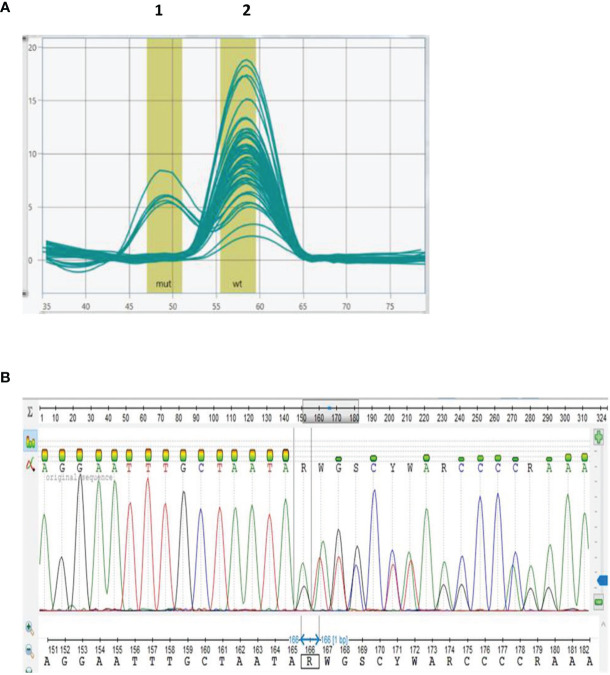
Genotyping of breast cancer patient samples using the BRCA 1.0 POC Research Assay designed to detect eight *BRCA1/2* founder/recurrent South African pathogenic variants. Melting curve analysis of *BRCA2* c.7934delG **(A)** with the CAL 560 probe indicating the presence of a second melting peak (peak 1) for six samples (five patients and the positive control). No pathogenic variants were detected in 45 DNA samples (peak 2). Detection of *BRCA2* c.7934delG was confirmed by Sanger sequencing **(B)**.

Once the BRCA 1.0 POC Research Assay’s analytical performance was confirmed, germline DNA of 50 BC patients was genotyped. Melt curve analyses indicated *BRCA2* c.7934delG in five of the patients **(****Figure 3A**). Detection of *BRCA2* c.7934delG (rs80359688) using the BRCA 1.0 POC Research Assay was confirmed by DNA sequencing for these patients **(****Figure 3B**). The electropherogram indicated a single base deletion, which resulted in a shift of the reading frame, prematurely truncating the associated peptide (**Figure 3B****)**. The BRCA 1.0 POC Research Assay initially failed partly for a single sample (1/50, 2%) as no results were obtained for four of the eight variants tested (two wells). Repeat of the assay resulted in successful genotyping of all eight *BRCA1/2* variants, indicating a user set-up error. Homozygous variant-negative (reference) samples generated a single melting peak (**Figures 2J, K****)**, whereas heterozygous variant-positive samples yielded two peaks (**Figures 2A–H**). All five *BRCA2* c.7934delG cases exhibited an additional melting peak at 50.0°C (**Figure 3A**).

The performance of the BRCA 1.0 POC Research Assay was further evaluated by analyzing the same samples using real-time PCR on the Roche LC480 real-time PCR instrument. Melting peak data were assessed manually to calculate melting peak temperatures for each of the eight *BRCA1/2* founder variants. All 50 samples were assigned the correct automated software calls using 2 ng to 62.5 pg of extracted input DNA. No false-negative or false-positive real-time PCR results were obtained with either the laboratory-based LightCycler or portable ParaDNA device using the same HyBeacon probes.

**Figure 4:**
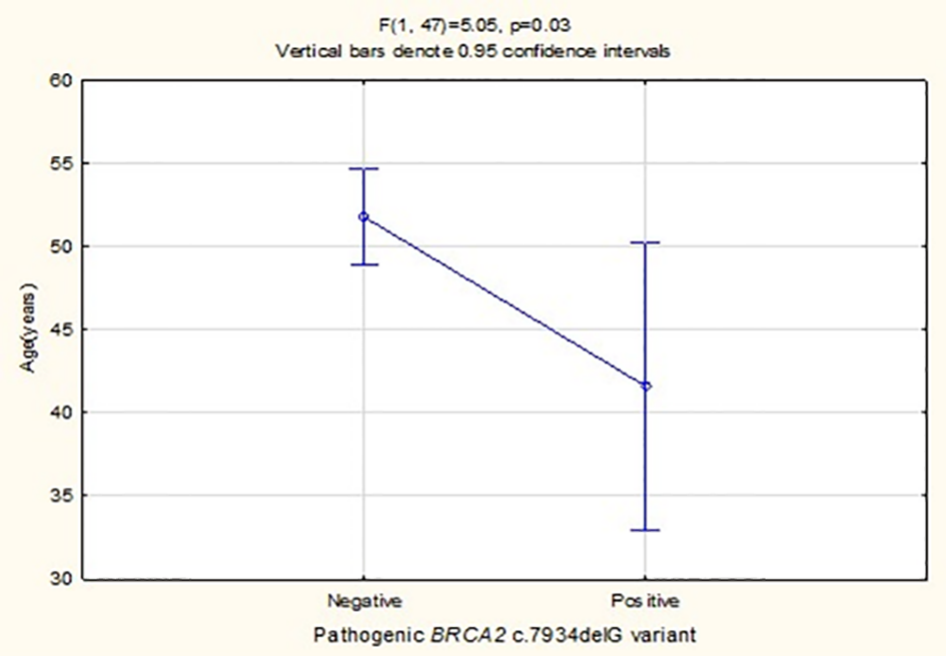
Comparison of the mean age between the five BC patients identified with *BRCA2* c.7934delG founder/recurrent pathogenic variant *versus* the 45 non-mutation carriers.

*BRCA2* c.7934delG was the only pathogenic variant detected in 10% of the 50 cases studied (n=5). Patients carrying this variant were diagnosed at a significantly younger age than variant-negative individuals (p=0.03), with mean ages of 41.60 ± 6.58 and 51.77 ± 9.83, respectively (**Figure 4**). Previous review of their histopathology reports indicated ductal/carcinoma of no special type in all five *BRCA2* c.7934delG carriers. Two cases had a low-risk MammaPrint profile for BC metastasis (luminal A) supporting omission of chemotherapy, whereas three had a high-risk profile (luminal B) as supported by molecular subtyping using the 80-gene BluePrint assay.

## Discussion

In this retrospective study, the results obtained with the rapid BRCA 1.0 POC Research Assay in patients with early-stage hormone receptor-positive BC showed a relatively high *BRCA1/2* founder variant detection rate (10%). This finding justifies screening of both familial and sporadic cases for germline *BRCA1/2* variants, and not only triple-negative BC when tumor type is considered in *BRCA1/2* risk prediction algorithms. Since the BRCA POC 1.0 Research Assay is inexpensive and can be manufactured locally, it may in future be utilized for all SA BC patients, irrespective of age, family history, ethnicity, tumor type or recurrence risk profile. Comparison with standard laboratory-based assays using stored DNA samples showed 100% concordance between the portable screening device and the various laboratory-based methods previously standardized. As the ParaDNA workflow is an integrated system from sample collection to result generation, separate DNA extraction can be eliminated in future with use of the sample collector also applied in forensics (23). Direct application using fresh cheek swabs/saliva as the preferred sample type performed excellent during the test development and optimization process (17). This makes POC testing using the ParaDNA device ideal for application of robust, first-tier targeted genetic tests in any clinic in Africa with access to personal or online genetic/genomic counseling support.

Africa is the second-largest continent, globally representing 14% of the world’s population (24). Although economic growth was stable between 2018 – 2019, the estimated 3.4% growth of countries such as SA, Egypt and Nigeria were below the decadal average of 5% for the continent. The number was predicted to increase to 3.9% during 2020, before the outbreak of the coronavirus disease 2019 (COVID-19) pandemic (25). The realization that Africa could benefit from the application of genomic medicine was captured in a policy paper composed by 38 researchers across the continent (26). This framework for the implementation of genomic medicine in Africa aims to reduce the disease burden by translating genomic research information into clinical application using PSGT as one of the proposed implementation strategies. With sufficient evidence for actionability, genomic medicine involving test panels such as our BRCA POC Research and COVID-19 screening assays using the same ParaDNA device, was fast-tracked in SA for test development and validation. Due to Africa’s extreme diversity, a “one size fits all” healthcare approach is not appropriate. By providing guidance, the WHO Regional Office for Africa aims to ensure that no one is left behind as the continent progresses towards sustainable and equitable health (27).

Implementing *BRCA1/2* targeted testing at POC is ideal for African countries for which an increased frequency of founder/recurrent actionable pathogenic variants have been identified through the years (28–30). For SA, the eight variants covered in the BRCA 1.0 POC Research Assay include three highly prevalent Ashkenazi Jewish/European founder variants of global relevance. *BRCA1* c.68_69delAG (rs80357914) has also been identified at an increased frequency in Egypt and Morocco. This highlights the value of the assay that can be adapted and redesigned according to each countries’ needs, depending on their familial BC mutation spectrum. *BRCA2* c.7934delG represents the most common SA founder variant. Therefore, it was not surprising that this single-base deletion was detected in our study cohort from a non-rural, private healthcare setting, at a 10% rate comparable to the 7.9% carrier status reported in the most extensive SA study published to date (151/1906) (18). Since *BRCA1/2* genetic testing could decrease mortality from breast, prostate, gynecological and some other cancers, and help inform therapy, there is a need to develop or adjust tools to enable targeted treatment and optimal care for all cancer patients (31). The updated pathology-adjusted Manchester score frequently used in SA for estimating the threshold for *BRCA1/2* probability (32), would be more effective in the SA population if patients with hormone receptor-positive BC (linked to the *BRCA2* c.7934delG founder variant in our study cohort) is considered for testing similarly to the inclusion of triple-negative BC. This will also apply to the use of other risk stratification tools such as CanRisk (https://www.phpc.cam.ac.uk/pcu/research/research-groups/cancer-group/canrisk/), which incorporates scientific discoveries in both cancer genomics and epidemiology. A genetic counseling toolkit enabling *BRCA1/2* founder/recurrent variant testing at the POC may add significant value, especially when incorporating the assessment of critical co-morbidities impacting on cancer risk and the option for NGS in eligible *BRCA1/2* founder variant-negative cases.

The new BRCA 1.0 POC assay can serve multiple purposes. Not only may patients and their close relatives become aware of being at increased risk of developing various cancer types, pathogenic *BRCA1/2* variants are also treatment targets for poly ADP-ribose polymerase (PARP) inhibitors (33). Clinical complications related to anti-cancer treatment regimens (34–36) furthermore led to the development of genomic assays such as the 70-gene MammaPrint microarray with level 1A evidence of clinical utility for prediction of chemotherapy benefit (37). Microarray analysis using tumor samples reformed our past understanding of BC as a single disease to a complex disorder consisting of at least four major subtypes, namely luminal A, luminal B, HER2-enriched and basal-type. Currently, tumor pathology including ER, PR and HER2 status is used routinely in SA as a proxy for identifying these subtypes, which may result in misclassification and inappropriate treatment in a subgroup of patients (20). While IHC assessment of ER, PR and HER2 status proved valuable in the private sector for selecting early-stage BC patients in SA for cost-effective use of the MammaPrint test generally performed on RNA extracted from surgical biopsies (19), post-surgery identification of a pathogenic *BRCA1/2* variant in germline DNA is a major concern. A bilateral mastectomy would be most effective in these cases as defective *BRCA1/2* genes increase the risk of a second breast primary, as well as other secondary cancers, which in turn are likely to metastasize and may be treatment-resistant (38–40). Although expensive, tumor gene profiling is currently reimbursed by private medical schemes in SA after careful patient selection using tools such as the MammaPrint pre-screen algorithm (19). The cost-benefit potential of selective MammaPrint testing was recently confirmed in a study of approximately 600 tumor samples of SA patients with early-stage BC, by employing a chemotherapy de-escalation strategy through clinical risk stratification (41). While toxicity profiles make hormone therapies an attractive option, the standard of healthcare on the African continent is reflected by the lack of ER, PR and HER2 assessment in many state institutions managing the disease (3, 42). This limitation was highlighted by Torrorey-Sawe et al. (3), as IHC was not determined to assess hormone receptor status in a relatively large proportion of study participants enrolled in a Kenyan whole exome sequencing study, initially focused on *BRCA1/2* for return of research results. This finding raised awareness for potential chemotherapy over-treatment in African patients with early-stage BC, which needs to be addressed in the future as part of the WHO’s development goals for the continent (27).

Although the small number of 50 study participants is a limiting factor, the samples available for this evaluation in patients with hormone receptor-positive breast cancer, are considered sufficient to support analytical validation and clinical utility of the BRCA 1.0 POC Research Assay as the primary aim achieved. Implementation of POC testing will decrease turn-around time and testing costs, as *BRCA1/2* founder variant testing at a reference laboratory in SA currently costs approximately ZAR 1500 to ZAR 2500, depending on the number of variants tested for according to ethnic/population group (43). In contrast, the BRCA 1.0 POC Research Assay’s projected cost once commercialized has been estimated at approximately ZAR 1000, with the option to adjust the design periodically to incorporate new actionable research data obtained for genes involved in hereditary breast and ovarian cancer syndrome. Regarding genetic counseling, a consultation session currently costs between ZAR 500 and ZAR 1300, depending on the duration. When performed in parallel with POC, the total cost should not exceed that of the current first-tier test alone (ZAR 2500). The BRCA POC Research Assay results will also help identify patients in need of more comprehensive hereditary breast and ovarian cancer screening using affordable NGS panels for *BRCA1/2* founder variant-negative patients (approximately ZAR 8000 in the state sector). As NGS analysis using an extended gene panel has been proposed to replace *BRCA1/2* founder variant testing in SA (43), the risk-benefit analysis recently performed helped pave the way forward (18). It provided insight into genetic professionals’ view for the future and confirmed the importance of a first-tier test now possible at POC.

**Figure 5:**
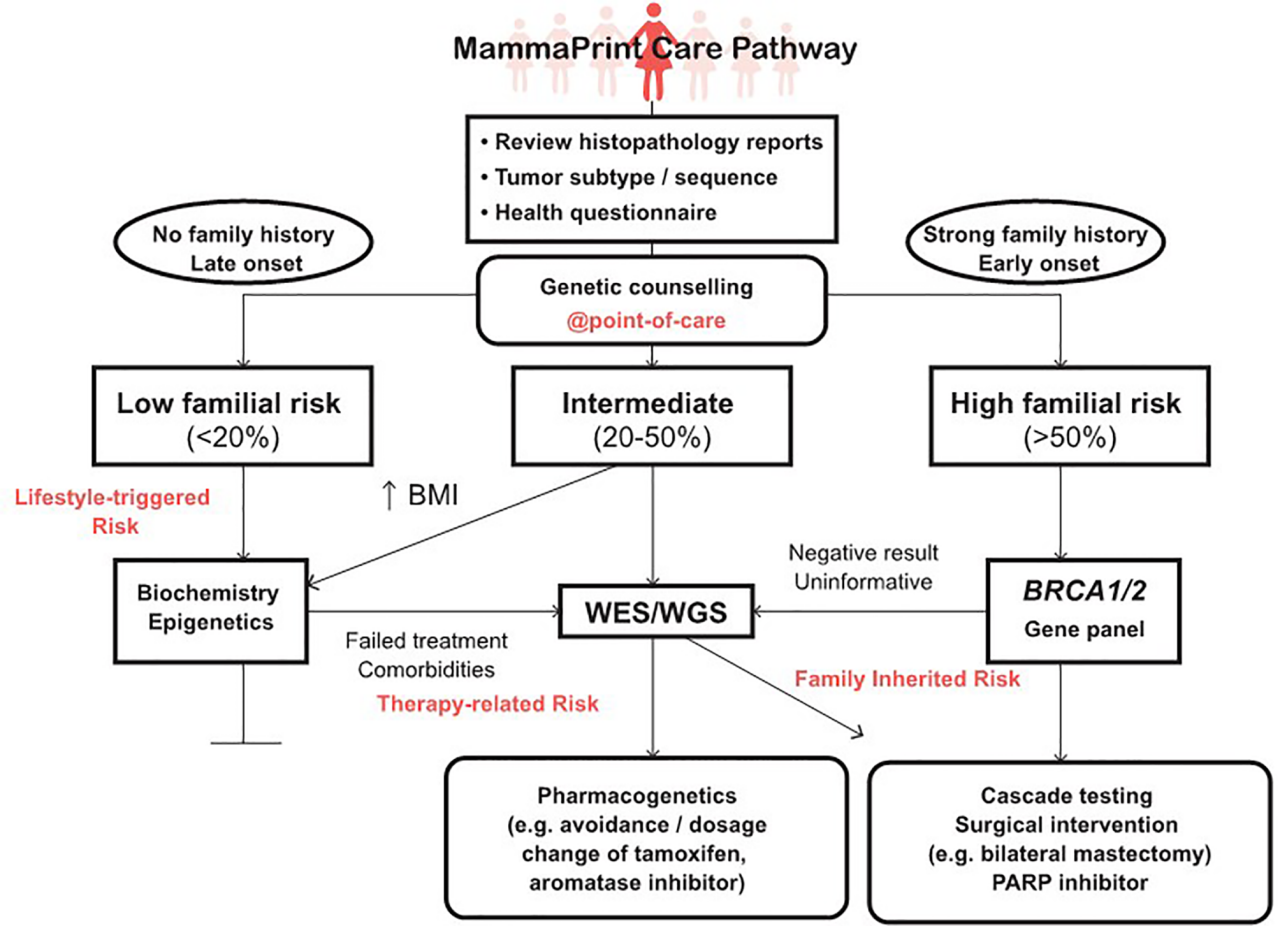
Incorporation of genetic counseling into the MammaPrint care pathway extending from point-of-care germline DNA testing to advanced genomic technologies, including clinical assessments such as body mass index (BMI) and whole exome/genome sequencing (WES/WGS) using a pathology-supported genetic testing approach.

The results obtained in this study supports incorporation of germline *BRCA1/2* testing early in the treatment planning of all BC patients in SA (18), including those opting for gene profiling using MammaPrint. Although MammaPrint is not available to BC patients in the public sector at present the prioritization of clinically high-risk patients for testing, such as those with node-positive (1–3) early-stage BC, could result in safe avoidance of chemotherapy and its associated side effects in approximately 50% of eligible BC patients (37, 41). By performing the BRCA 1.0 POC Research Assay in conjunction with transcriptional gene profiling, unnecessary medical expenditure may be further reduced. Therefore, health economic studies are warranted to determine potential cost-benefits from performing genetic counseling combined with rapid *BRCA1/2* POC testing, compared to usual care. By pro-actively positioning POC testing as a genetic counseling tool, we envisage a future where BC patients will have access to personalized genomic medicine across the continuum of cancer care, as illustrated in **Figure 5**. All BC patients will benefit from genetic counseling at the POC, as *BRCA1/2* variant carriers with a high-risk MammaPrint 70-gene profile are at increased risk for both local and distant/metastatic recurrence of their cancer. Those patients with a low-risk transcriptional gene profile may be unaware of the presence of a possible *BRCA*/other germline variants unrelated to risk for metastasis assessed by MammaPrint. This was evidenced by delayed detection of the *BRCA2* c.7934delG variant (initially in tumor DNA using NGS) in one of our study participants diagnosed with metachronous bladder cancer four years after receiving a low-risk MammaPrint result (44). Integration of germline DNA testing and tumour genetics is therefore essential for optimal treatment of patients at increased risk of secondary cancers and BC recurrence (45, 46). Validation of genomic medicine test panels and transfer of actionable gene variants to POC devices offers a flexible platform for adding modifiable environmental exposure data to inform intervention and prevention efforts towards global health (47–49). For genomic medicine to become a reality in Africa, a screening algorithm standardized by the Department of Health needs to be implemented to ensure adherence to set standards for optimal care provided to all BC patients (50).

In conclusion, this study is the first to comprehensively investigate the cost-saving potential and clinical value of *BRCA1/2* POC testing. By using HyBeacon probe technology, the *BRCA1/*2 test was transferred from a laboratory-based assay requiring sample transport (extra cost and risk of sample mix-up) and batching (expensive as multiple control reactions are required) to a rapid, robust assay performed at POC on the portable ParaDNA device. By performing targeted genotyping by trained healthcare professionals as a first-tier test at the POC in parallel to genetic counseling as a feasible option for all histologically confirmed breast/ovarian cancer patients, sample collection and testing can be moved out of a tertiary healthcare setting to currently unreached communities. This will reduce loss to follow-up and create the ability to improve care by delivering on-demand psychosocial support directly to the patient and indirectly to the community, where needed. Our findings provided proof of the BRCA 1.0 POC Research Assay’s analytical performance, while the clinical utility was evidenced by reaching the 10% threshold for cost-effective variant detection in patients with hormone receptor-positive BC, not currently considered for routine *BRCA1/2* testing in SA. Once regulatory authorities have approved on-site BRCA POC testing, this model may be presented to policymakers for wider implementation of oncogenomic medicine in Africa.

## Data Availability Statement

The raw data supporting the conclusions of this article will be made available by the authors, without undue reservation. The clinical characteristics of the 50 breast cancer patients screened were previously reported by the first author at http://etd.cput.ac.za/handle/20.500.11838/3080.

## Ethics Statement

The studies involving human participants were reviewed and approved by the Health and Wellness Sciences Research Ethics Committee of the Cape Peninsula University of Technology in Cape Town (CPUT/HW-REC 2018/H10), the Ethics Committee of the Faculty of Health Sciences, University of the Free State (UFS-HSD2019/1835/291001), and approved as a sub-study under reference number N09/06/166 by the Health and Research Ethics Review Committee of Stellenbosch University, SA. The patients/participants provided their written informed consent to participate in this study.

## Author Contributions

LM, KG, and MJK made substantial contributions to the conception, design and completion of this project involving both germline and tumor genetics. LM obtained ethics approval, selected the data for analysis and performed the genetic studies together with KM, AP, and DF. DF developed and manufactured the assay used in this study and provided training and expert guidance throughout the project’s conception and execution. RT-S reviewed the histopathology reports, and MK performed the statistical analysis. FP and PE provided clinical oversight and verified genetic results obtained in this study against their patients’ positive and negative test results reported elsewhere. NM obtained inter-institutional ethics approval, verified the analytical assay validation, and framed the study in relation to past achievements and the present standard of care. All authors contributed to the article and approved the submitted version.

## Funding

The research reported in this publication was supported by the Strategic Health Innovation Partnerships Unit of the South African Medical Research Council (SAMRC), with funds received from the South African Department of Science and Innovation (S003665, S006652) and the Cancer Association of South Africa (CANSA). The South African BioDesign Initiative of the Department of Science and Innovation and the Technology Innovation Agency are acknowledged for funding part of this research (grant number 401/01). RT-S received a two-year postdoctoral fellowship from Stellenbosch University. Any opinion, findings and conclusions, or recommendations expressed in this article are those of the authors and the funders accept no liability for the content of the article.

## Conflict of Interest

Author DF was employed by company LGC Limited. MJK is a non-executive director and shareholder of Gknowmix (Pty) Ltd. that is involved with the development of the POC 1.0 BRCA Research Assay.

The remaining authors declare that the research was conducted in the absence of any commercial or financial relationships that could be construed as a potential conflict of interest.

## Publisher’s Note

All claims expressed in this article are solely those of the authors and do not necessarily represent those of their affiliated organizations, or those of the publisher, the editors and the reviewers. Any product that may be evaluated in this article, or claim that may be made by its manufacturer, is not guaranteed or endorsed by the publisher.
